# Multi-scale diffusion model for underwater image restoration and enhancement

**DOI:** 10.1371/journal.pone.0331465

**Published:** 2025-09-10

**Authors:** Yuan Fang, Qianyi Li, Kai Wang

**Affiliations:** 1 School of Innovation and Entrepreneurship, Dalian Polytechnic University, Dalian, Liaoning, China; 2 School of Information Science and Engineering, Dalian Polytechnic University, Dalian, Liaoning, China; 3 The Institute of Port Information Digitalization, China Liaoning Port Group Co. Ltd., Dalian, Liaoning, China; Nanyang Technological University, SINGAPORE

## Abstract

**Background:**

Underwater environments face challenges with image degradation due to light absorption and scattering, resulting in blurring, reduced contrast, and color distortion. This significantly impacts underwater exploration and environmental monitoring, necessitating advanced algorithms for effective enhancement.

**Objectives:**

The study aims to develop an innovative underwater image enhancement algorithm that integrates physical models with deep learning to improve visual quality and surpass existing methods in performance metrics.

**Methods:**

The proposed method employs a multi-level design combining physical insights from the diffusion model with deep learning. It utilizes an encoder-decoder pipeline for image decomposition, adaptive color adjustment through a color encoder network, and pixel optimization using an inverse denoising diffusion model to achieve enhanced visual quality.

**Results and conclusion:**

Experimental results demonstrate significant improvements, with an average increase of 6.61 in PSNR, 0.15 in SSIM, and 0.87 in UIQM. The integration of physical models and deep learning proves effective for underwater image enhancement. This research advances the field by providing a robust framework for addressing imaging challenges in underwater environments.

## Introduction

Underwater image processing plays a crucial role in acquiring oceanic information and is widely applied in marine ecology research, underwater archaeology, and marine monitoring. It enhances situational awareness by integrating human expertise, machine intelligence, and physical-environmental dynamics. However, complex underwater environments—characterized by light scattering, wavelength-dependent attenuation, and turbidity—lead to severe visual data degradation, significantly hindering autonomous decision-making [1]. The absorption and scattering effects of the water medium, along with interference from suspended particles, result in substantial image quality degradation, manifesting as color distortion, low contrast, blurring, and insufficient brightness. These issues not only impair visual quality but also pose significant challenges for computer vision tasks such as underwater image segmentation and object detection. Consequently, enhancing underwater image quality and restoring visual clarity have become critical research challenges in marine science and underwater exploration.

The primary factors contributing to underwater image degradation include particle scattering and light absorption in seawater, which reduce image clarity and introduce color shifts, low contrast, and blurring [2]. These distortions severely affect computer vision performance, making underwater image enhancement essential for obtaining clear and reliable information. Traditional enhancement approaches often rely on physical models that estimate medium transmission maps and atmospheric light to improve image quality. However, these methods frequently suffer from inaccurate degradation modeling and fail to achieve satisfactory results in complex underwater conditions. In recent years, deep learning has provided a breakthrough in underwater image enhancement. Techniques such as convolutional neural networks (CNNs) and generative adversarial networks (GANs) have demonstrated the capability to learn nonlinear mappings between degraded and clear images, achieving significant improvements across various underwater tasks [3].

Despite these advancements, several challenges remain. Many deep learning-based methods depend on paired datasets for training, but acquiring high-quality reference images for underwater environments remains a major obstacle. Additionally, existing enhancement techniques often struggle to simultaneously address multiple degradation factors. For example, improving brightness may introduce additional noise, while enhancing color fidelity could compromise texture details [4]. Furthermore, many approaches heavily rely on prior knowledge and lack adaptability to varying environmental conditions. To address these issues, this study employs a diffusion model for unsupervised learning, integrating the advantages of physical modeling and deep learning. A multi-level enhancement strategy is designed to refine image details and overcome the limitations of current methods [5].

The key contributions of this paper are twofold: (1) leveraging the diffusion model’s capability to simulate complex data distributions and generate high-quality underwater images, and (2) decoupling degradation factors in the frequency domain to enhance each aspect of image quality, thereby effectively addressing the challenge of handling multiple degradation factors simultaneously.

## Related work

### Underwater image enhancement techniques

In 1994, Zuiderveld K first proposed the Contrast Limited Adaptive Histogram Equalization (CLAHE) method, a classical image enhancement technique that holds a significant position in the field. In 2011, Han J.H., Yang S., and Lee B.U. introduced a new 3D color histogram equalization method that produces a uniform 1D grayscale histogram [6]. These methods enhance image quality by adjusting pixel values, but they do not account for the various factors causing image degradation. Although traditional CLAHE methods can improve image contrast, they may also introduce noise, especially in underwater images, which are complex and often noisy. The hybrid CLAHE algorithm designed by Hitam et al. optimized the processing pipeline, successfully avoiding the common noise amplification issue inherent in traditional CLAHE methods, thereby proving more efficient for underwater image enhancement. Naik and his team proposed a shallow neural network model, which, compared to traditional deep neural network (DNN) models, requires fewer parameters and exhibits higher computational efficiency while still maintaining strong performance. This model has demonstrated good generalization capabilities in enhancement tasks, showing its effectiveness in various types of images, making it highly adaptable in practical applications like underwater image enhancement. Currently, underwater image enhancement methods can be broadly classified into two categories: physical model-based approaches and deep learning-based techniques. In the field of underwater image enhancement, physical model-based methods restore degraded underwater images by estimating medium transmission characteristics and atmospheric light to enhance visual effects and improve overall image quality. For example, the color recovery model proposed by Kang attempts to compensate for the effects of absorption and scattering by the water body on light of different wavelengths, thus recovering the scene’s true colors [7]. D.L. Che et al. proposed an underwater image enhancement method based on light estimation, which, in combination with the scattering model of underwater images, estimates depth and lighting conditions to restore image clarity and contrast [8]. Common deep learning-based methods typically use Convolutional Neural Networks (CNNs) to perform tasks such as dehazing, denoising, and color correction to improve image quality. For instance, GAN-based image enhancement networks can effectively reconstruct high-quality underwater images, especially when dealing with low-contrast and color-distorted images [9]. CNN-based underwater image enhancement methods, such as the Deep Underwater Image Enhancement Network (DUINet), optimize and enhance underwater images through an end-to-end training mechanism. However, compared to physical model-based methods, deep learning approaches often heavily rely on the distribution of training data, and any distribution change in the underwater image during inference can lead to a significant degradation in enhancement performance.

### Denoising diffusion probabilistic models

The Denoising Diffusion Probabilistic Model (DDPM), proposed by Ho et al. in 2020, forms the foundation of diffusion models. It generates samples by gradually adding noise to data and learning how to remove the noise through a reverse process [10]. To improve sampling efficiency, the Denoising Diffusion Implicit Model (DDIM), introduced by Song et al. in 2020, made key optimizations to the DDPM. DDIM introduces a non-Markov diffusion process, significantly accelerating the sampling process of diffusion models [11]. To make the diffusion model capable of generating more controllable images, conditional information can be incorporated into the model, enabling conditional generation. This method allows the diffusion model to generate images under specific conditions, such as incorporating label information, text descriptions, or other conditional constraints. Dhariwal et al. [12] proposed a classifier-guided training strategy within the diffusion model, which adjusts the conditional information during the generation process. By using the gradient of a pre-trained classifier, this approach conditions each step of the generation process, ensuring that the generated samples adhere to the specified conditions. Benefiting from conditional generation, diffusion models have been successfully applied to various image restoration tasks, including denoising, deblurring, and inpainting. For example, in the task of underwater image enhancement, Tang et al [13]. utilized the generative capabilities of diffusion models to design a lightweight architecture that improves image quality and detail recovery.

## Methodology

This section primarily introduces the framework and details of the image enhancement network, which is designed to restore degraded underwater images while preserving texture details and ensuring color consistency. The network first employs frequency-domain processing to more effectively identify and differentiate various degradation factors, enabling targeted enhancement strategies. The objective of this network is to transform an input degraded underwater image $x\in R^{B\times C\times H\times W}$ into its corresponding clear image $y^{\prime }\in R^{B\times C\times H\times W}$, where *B*, *H*, *W*, and *C* represent the batch dimension, height, width, and number of channels, respectively. The optimized clear image $y^{\prime }$ is then fed into a diffusion model to generate a higher-quality output image $y\in R^{B\times C\times H\times W}$.

### Network architecture

First, the input image $x\in R^{32\times 3\times 256\times 256}$ is obtained and initially enhanced to produce the real underwater image $x_{0}\in R^{32\times 3\times 256\times 256}$. The process first obtains an output of shape 32× 64 × 256 × 256 for each group through multi-scale dilated convolution groups,then it is obtained by feature fusion an output of shape 32×64×256×256. The enhanced underwater image *x*_0_ is then mapped into the feature space through a convolutional layer. Next, these features are fed into an encoder-decoder network for processing, where most of the computations are performed by the spatial-frequency blocks. This module consists of downsampling and upsampling operations, with each upsampling layer having a shortcut connection to the corresponding level of the encoder. Finally, a coarse output $y_{0}\in R^{32\times 1\times 64\times 64}$ is generated.

Meanwhile, the initial image *x*_0_ is also fed into a color encoder. The color encoder network extracts visual features from the image and subsequently maps the extracted features to different scales for adaptive color querying of image features. Next, the coarse output image $y_{0}\in R^{32\times 1\times 64\times 64}$ is combined with the generated color features to obtain a vivid output image $y^{\prime }\;\in \;R^{32\times 3\times 256\times 256}$. Finally, the best matching natural image *x* is found by searching in the domain of natural image R, and the final generated image $y\;\in \;R^{32\times 3\times 256\times 256}$ is generated by the inverse denoising model.

In addressing the problem of underwater image enhancement, decoupling degradation factors in the frequency domain. Such as color distortion, low contrast, and blurring has proven to be an effective strategy. Frequency domain processing enables clearer identification and differentiation of these degradation factors, allowing for targeted enhancement approaches. However, a straightforward application of the Fast Fourier Transform (FFT) to decompose an image into its amplitude and phase components, followed by an Inverse Fast Fourier Transform (IFFT) with amplitude and phase swapping, introduces additional artifacts such as shadows and noise [14].

Alternatively, decomposing an image into phase and amplitude components via FFT and subsequently reconstructing a new image using IFFT can provide further insights based on prior knowledge. Specifically, images reconstructed from phase information predominantly preserve texture and structural details, whereas those reconstructed from amplitude components primarily retain color and illumination details, while completely losing texture and structure. This distinction underscores the necessity of carefully designing frequency-domain processing techniques to achieve effective underwater image enhancement.

In this work, A network is designed to ensure that the enhanced image exhibits improved color fidelity, increased brightness, minimized noise, and enriched texture details. The fundamental processing module adopts a three-path architecture, consisting of an initial enhancement path, a color correction path, and a deblurring path.

**Fig 1:**
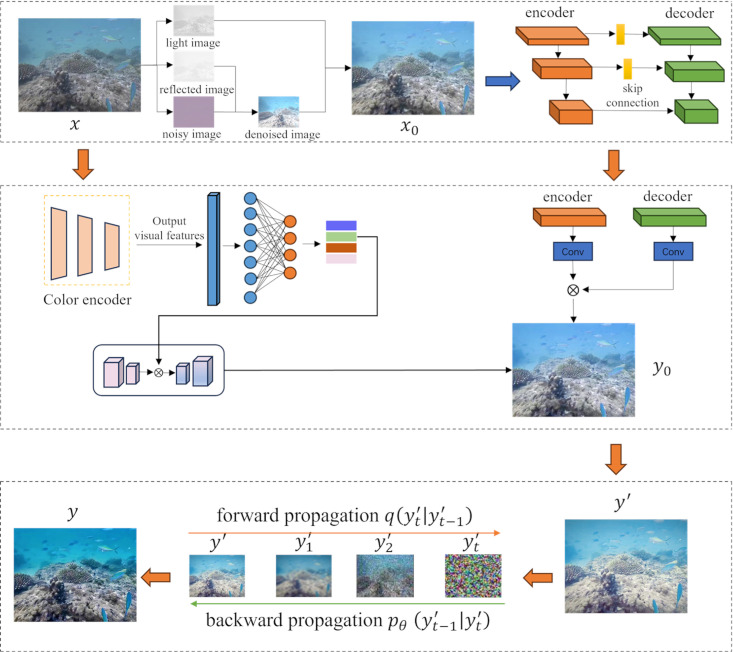
Network structure diagram.

**Fig 2:**
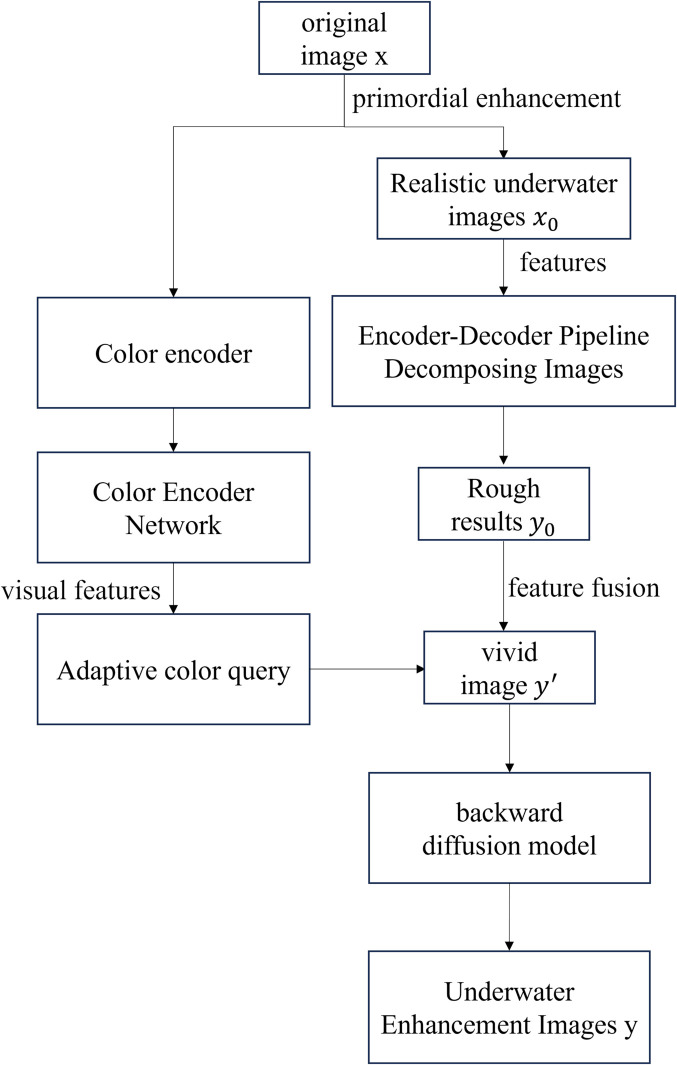
Network flowchart.

The initial enhancement path aims to perform preliminary enhancements on the input image to improve its overall quality. This is achieved by adjusting fundamental parameters such as contrast and brightness, thereby increasing the visibility of the image. The color correction path further refines the image through deep processing, enhancing spatial details and structural information to achieve better perceptual quality. Meanwhile, the deblurring path utilizes a diffusion-based structure to refine the image, effectively recovering texture and structural details while optimizing color, brightness, and noise characteristics. The network structure and flowchart are shown in Figs 1 and 2.

### Module implementation

#### Initial enhancement module.

Various underwater image enhancement techniques have been proposed, including methods that decompose color, brightness, texture, and other image attributes in the frequency domain [15]. However, raw underwater images often suffer from noise, low color fidelity, and poor contrast, which can negatively impact the enhancement process. To address these issues, a preliminary enhancement step is necessary to suppress noise and improve the overall image quality before further processing. This preliminary enhancement improves color fidelity, increases brightness, reduces noise, and enriches texture details.

**Fig 3:**
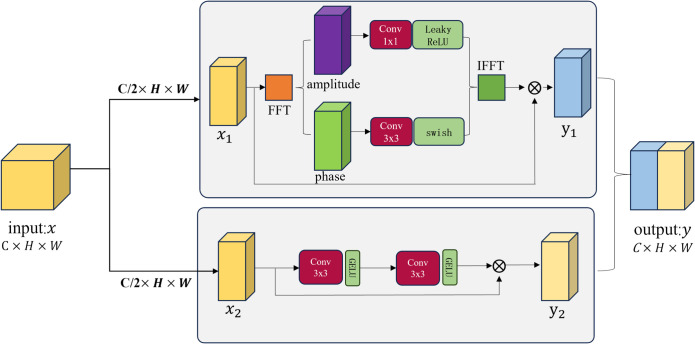
Preliminary enhancement module diagram.

As illustrated in Fig 3, the image undergoes a preliminary enhancement stage, followed by feature extraction using an encoder-decoder pipeline similar to the traditional U-Net structure [16]. In this framework, the input features are partitioned into two components: (placeholder for specific terms). The image is then transformed from the spatial domain to the frequency domain via the Fast Fourier Transform (FFT), decomposing it into magnitude and phase components. The transformation is mathematically represented in Eq (1).

$$F\left(x_{1}\right)=FFT\left(x_{1}\right)=M\left(x_{1}\right)+i\cdot P\left(x_{1}\right)$$ (1)

where, *x*_1_ is the first half of the input signal along the channel dimension. *FFT*(*x*_1_) is a fast Fourier transform of *x*_1_ to convert it from the spatial domain signal to a complex signal in the frequency domain. *M* is magnitude component, *P* is the phase component extracted from the transformed image.

The magnitude and phase components are processed separately. The magnitude component undergoes convolution using a 1 × 1 convolutional kernel to preserve the structural integrity of the magnitude information and prevent distortion caused by excessively large kernels.

To enhance the magnitude features, the LeakyReLU activation function is applied. During network training, LeakyReLU aids in retaining more detailed information, thereby improving the model’s ability to capture and represent complex data features. This enhances the network’s capability to process intricate information effectively. The transformation process is mathematically represented in Eq (2).

$$M^{\prime }\left(x_{1}\right)=LeakyReLU\left(Conv_{1\times 1}\left(M\left(x_{1}\right)\right)\right)$$ (2)

The phase component *P* plays a crucial role in preserving the shape and details of the image. Therefore, a 3 × 3 convolutional kernel is applied to process the phase information. During the model construction, the Swish activation function is employed to enhance the non-linear representation capability. By doing so, the model can more effectively maintain the structural details of the image while processing, thereby improving the effectiveness of feature extraction. The transformation process is mathematically represented in Eq (3).

$$P^{\prime }\left(x_{1}\right)=Swish\left(Conv_{3\times 3}\left(P\left(x_{1}\right)\right)\right)$$ (3)

After processing both the magnitude and phase components, the inverse fast Fourier transform (IFFT) is applied to convert the data back to the spatial domain. This transformation is mathematically represented in Eq (4).

$$x_{1}^{\prime }=IFFT\left(M^{\prime }\left(x_{1}\right)+i\cdot P^{\prime }\left(x_{1}\right)\right)$$ (4)

The enhanced magnitude and phase components are connected to the original input *x*_1_ through a residual connection, resulting in the final output feature *y*_1_. This process is mathematically represented in Eq (5).

$$y_{1}=x_{1}+x_{1}^{\prime }$$ (5)

For *x*_2_, convolution operations are directly performed in the spatial domain using two 3 × 3 convolutional layers, each followed by a GELU activation function to enhance spatial features. The GELU activation function smooths nonlinear transformations, thereby improving feature detail enhancement and enabling the model to better learn and express data features. Finally, the output of the spatial pathway is connected to *x*_2_ via a residual connection to obtain *y*_2_. This process is mathematically represented in Eqs (6) and (7).

$$x_{2}^{\prime }=GELU\left(Conv_{3\times 3}\left(GELU\left(Conv_{3\times 3}\left(x_{2}\right)\right)\right)\right)$$ (6)

$$y_{2}=x_{2}+x_{2}^{\prime }$$ (7)

Where *x*_2_ is the second half of the input signal along the channel dimension. By employing a concatenation operation, *y*_1_ and *y*_2_ are merged to obtain the final output feature *y*. This feature fusion strategy enables the model to leverage both frequency-domain and spatial-domain information simultaneously, thereby enhancing feature representation capabilities and facilitating the disentanglement of degradation factors. This process is mathematically expressed in Eq (8).

$$y=Concat\left(y_{1},y_{2}\right)$$ (8)

#### Color correction module.

The issue of color attenuation in underwater imaging presents another significant challenge. To address this, previous studies have proposed various prior-based solutions. For instance, some methods rely on the dark channel prior (DCP), while others utilize underwater scene priors or histogram distribution priors for processing [17]. However, due to the complexity of underwater environments, the degree of color attenuation varies, and a single prior-based approach cannot effectively handle all color cast and attenuation issues.

Therefore, in the color correction module, we design a color encoder network is designed to extract visual features from the image. The extracted features are then mapped to different scales to enable adaptive color queries for image characteristics. The color encoder network consists of multiple convolutional layers and downsampling operations, which progressively extract comprehensive image features through hierarchical downsampling. These multi-scale features are subsequently used to optimize color queries.

Additionally, this module employs a color encoder to compute the color histogram of the image in the RGB color space. The color histogram represents the color distribution of the image by counting the frequency of specific pixel intensities or intensity intervals. Given an image *x*, the pixel value at a given position is denoted as Eq (9).

x(i,j)=(R(i,j),G(i,j),B(i,j)) (9)

where, R(i,j),G(i,j),B(i,j) represent the pixel values in the red, green, and blue channels, respectively. For each color channel (i,j), the pixel values are segmented into *k* intervals, as shown in Eq (10).

$$Bin_{c}^{k}=\left\{v|v\in \left(k\cdot \Delta ,\left(k+1\right)\cdot \Delta \right)\right\},k=0,1,\cdots K-1$$ (10)

where, Δ represents the width of each interval, typically set as $\Delta =\frac{255}{k}$.

To compute the number of pixels in each channel *c* that belong to each interval $Bin_{c}^{k}$, the calculation is given by Eq (11).

$$H_{c}\left[k\right]=\sum_{i,j}1\left(I_{c}\left(i,j\right)\in Bin_{k}^{c}\right)$$ (11)

where, $H_{c}\left[k\right]$ represents the frequency of the *k*-th interval in the color histogram for channel *c*, and 1(·) is an indicator function that equals 1 if the condition holds, and 0 means otherwise.

**Fig 4:**
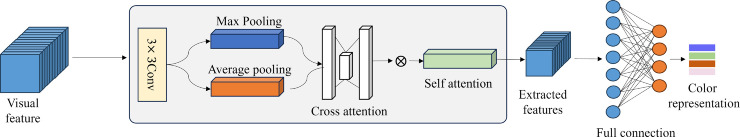
Color correction module network structure.

As shown in Fig 4, during the training process, the color encoder is utilized to extract color features consistent with the reference image. The extracted features are then mapped to different scales for adaptive color querying of image features. Furthermore, connections are established between cross-attention,

#### Deblurring module.

Since the vivid output image $y^{\prime }$ contains additive Gaussian noise, the deblurring module is used to remove more than the noise to improve the structure definition and restore the lost texture. The deblurring task in this paper is realized based on the inverse denoising diffusion model. (Diffusion Models). Diffusion models generate images through a forward diffusion process and a reverse diffusion process [18]. The purpose of this module is to process blurred images and transform them into sharper images through a series of algorithms and operations, thereby improving the image quality. The key idea behind the diffusion model is to progressively add noise to the original image (the forward diffusion process) and then learn how to gradually recover the image from the noise (the reverse diffusion process) [19].

During the forward diffusion process, noise is gradually added to the image $y^{\prime }$, obtained from the previous two modules, to simulate the blurring or degradation process. The forward diffusion process progressively transforms the image $y_{0}^{\prime }$ into pure noise $y_{t}^{\prime }$ over multiple time steps *t*.

In the deblurring task, the blurry image is obtained through a similar noise addition process. This process is formulated as shown in Eq (12).

$$y^{\prime }=\sqrt{\alpha_{t}}y_{t-1}^{\prime }+\sqrt{1-\alpha_{t}}\epsilon_{t},t=1,2,\cdots T$$ (12)

where, $y_{t-1}^{\prime }$ represents the image at step *t*, $\alpha_{t}$ is a factor between 0 and 1 that controls the intensity of the noise. $\epsilon_{t}$ is Gaussian noise, and *T* denotes the maximum time step in the diffusion process.

A general the reverse diffusion process learns how to gradually recover the original image from pure noise. This process uses a neural network model to predict the noise at the current time step t, but there is no clear reference image to train a deep model to control its diffusion direction. Therefore, this module uses the natural image domain as a condition to control the diffusion direction, so as to generate higher quality images. To recover the data from the noise, each step of the reverse process can be defined as shown in Eq (13).

$$p_{\theta }\left(y_{t-1}^{\prime }|y_{t}^{\prime }\right)=N\left(y_{t-1}^{\prime };\mu_{\theta }\left(y_{t}^{\prime },t\right),\sigma_{t}^{2}I\right)$$ (13)

where, $p_{\theta }\left(y_{t-1}^{\prime }|y_{t}^{\prime }\right)$ represents the distribution that predicts the previous time-step image $y_{t-1}^{\prime }$ from the current image $y_{t}^{\prime }$; $\mu_{\theta }\left(y_{t}^{\prime },t\right)$ is the mean predicted by the model,used to generate $y_{t-1}^{\prime }$. And $\sigma_{t}^{2}I$ denotes the noise scale in the reverse diffusion step. To perform conditional generation,the inverse procedure conditioned on natural images can be rewritten as shown in Eq (14).

$$\log p_{\theta }(y_{t-1}^{\prime }|y_{t}^{\prime },c)=\log\left(p_{\theta }(y_{t-1}^{\prime }|y_{t}^{\prime })p_{\phi }(c|y_{t}^{\prime })\right)+C_{1}$$ (14)

where $p_{\theta }(y_{t-1}^{\prime }|y_{t}^{\prime })$ is an unconditional denoising model. $p_{\phi }(c|y_{t}^{\prime })$ is the image condition term, which measures how well the current noise image x matches the target condition image c. *C*_1_ is the normalization constant.

The next goal is to generate the augmented image $y^{\prime }$ by searching in the domain of the natural image *c* to find the best matching natural image *c*. The first step is to adjust the mean calculation to include the conditional gradient. The process can be defined as shown in Eq (15)

$$\mu^{\prime }=\mu_{0}(y_{t}^{\prime },t)+s\cdot \Sigma \cdot \nabla_{y_{t}}\log p_{\phi }(c|y_{t}^{\prime })$$ (15)

Where *s* is the guiding strength and Σ is the diffusion model variance. At generation step *t*, sampling $y_{t}^{\prime }$, the conditional posterior probability is approximated as follows Eq (16).

$$p_{\theta }(y_{t-1}^{\prime }|y_{t},c)\propto p_{\theta }(y_{t-1}^{\prime }|y_{t})\cdot p_{\phi }(c|y_{t}{)}^{s}$$ (16)

**Fig 5:**
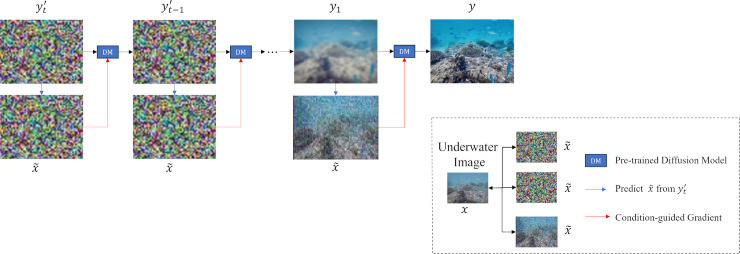
Deblurring module network structure.

By adjusting the intensity *s*, the fidelity and condition consistency of the generated image are controlled. $y^{\prime }$ is transformed to align with *c* in the natural image domain and compute the similarity between the generated image and the natural image as the matching function $\ell_{match}(c,y^{\prime })$. The network structure of the deblurring module is shown in Fig 5.

#### Loss function.

For the preliminary enhancement module loss aims to ensure the rationality of the initial image decomposition and the stability of the diffusion denoising. The loss function of the augmentation module is defined as follows Eq (17).

$${ℒ}_{enhance}={ℒ}_{recon}+{ℒ}_{diff-prior}+{ℒ}_{smooth}$$ (17)

where ${ℒ}_{recon}$ is decomposition reconstruction loss, ${ℒ}_{diff-prior}$ is diffusion prior loss, ${ℒ}_{smooth}$ is illumination smoothness constraints.

The color correction module loss is based on color histogram analysis and adversarial training. The loss function of the color correction module is defined as follows Eq (18)

$${ℒ}_{color}={ℒ}_{hist}+{ℒ}_{decouple}+{ℒ}_{adv-color}$$ (18)

where ${ℒ}_{hist}$ is histogram of the bulldozer distance, ${ℒ}_{decouple}$ is chrominance-luminance decoupling loss, ${ℒ}_{adv-color}$ is adversarial color loss.

The defuzzification module loss is designed by combining conditional diffusion with perceptual optimization. The loss function of the defuzzification module is defined as follows Eq (19)

$${ℒ}_{deblur}={ℒ}_{elbo}+{ℒ}_{guide}+{ℒ}_{percep}$$ (19)

where ${ℒ}_{elbo}$ is variational lower bound loss, ${ℒ}_{guide}$ is conditional gradient guided loss, ${ℒ}_{percep}$ is perceptual sharpness loss.

In order to ensure that these modules maintain the consistency of the output when they work together and avoid the conflict of the optimization objectives of each module, the cross-module consistency loss is designed as follows Eq (20).

$${ℒ}_{consist}={ℒ}_{freq}+{ℒ}_{multi-scale}$$ (20)

where ${ℒ}_{freq}$ is alignment loss in the frequency domain, ${ℒ}_{multi-scale}$ is multi-scale pyramid loss.

In order to meet the multi-task collaborative optimization strategy, a hierarchical loss function is designed to cover the joint training requirements of three sub-modules: image enhancement, color correction and deblurring. The total loss function is defined as follows in Eq (21).

$${ℒ}_{total}=\lambda_{1}{ℒ}_{enhance}+\lambda_{2}{ℒ}_{color}+\lambda_{3}{ℒ}_{deblur}+\lambda_{4}{ℒ}_{consist}$$ (21)

where $\lambda_{1}$, $\lambda_{2}$, $\lambda_{3}$ and $\lambda_{4}$ are hyper-parameters to balance the contribution of each loss.

### Experiment results analysis

#### Dataset.

The constructed dataset consists of real underwater images captured using an underwater camera at a port and dock in Dalian, Liaoning Province. Initially, 1000 real underwater images were extracted from video recordings. Among these, images with different types of degradation were selected, including 300 images with color cast, 200 images with blurriness, and 260 images with low illumination.

The test dataset is divided into two parts: Test1 and Test2. Test1 contains 760 underwater images exhibiting the three types of degradation. Test2 consists of the remaining 240 underwater images without significant degradation.

#### Experiment settings.

To introduce enhance the robustness of our model, we incorporate horizontal flipping and rotation were employed during training. To ensure a fair comparison, all images were resized to 256 × 256 during both training and testing. For the hyperparameters of our model, the weights $\lambda_{1}=1.0$, $\lambda_{2}=0.8$, $\lambda_{3}=1.2$, $\lambda_{4}=0.5$ in Eq (21). Other parameters adhere to the default settings in PyTorch. We trained them for 100 epochs with a batch size of 32 with a learning rate $1\times 10^{-4}$.

### Comparative experiments

#### Quantitative evaluation results.

In order to avoid the deviation of experimental results caused by the difference of input image quality. Before evaluating the performance of the different methods,the original images of all the test sets were evaluated for no-reference quality by UIQM, NIQE, and QIPE to ensure objective consistency of the input degradation degree.

To evaluate the performance of different methods, both quantitative and subjective visual quality assessments were conducted on the Test1 dataset. The evaluation metrics used for Test1 include Structural Similarity Index (SSIM), Peak Signal-to-Noise Ratio (PSNR), Natural Image Quality Evaluator (NIQE), and Perceptual Image Quality Evaluator (PIQE) to measure the difference between the enhanced images and the reference images [20–22]. Additionally, the Underwater Image Quality Metric (UIQM) was introduced to evaluate the intrinsic quality of the images.

SSIM measures the similarity of two images in three dimensions: brightness, contrast and structure. The value of SSIM is in the range of [−1, 1], the closer the value is to 1, the more similar the images are; PSNR is based on the mean square error (MSE) to evaluate the distortion of the image. Higher values of PSNR indicate better image quality; NIQE is a non-referenced image quality evaluation metric that measures the naturalness of an image; lower NIQE scores indicate that the image is closer to a natural image; PIQE is a reference-free image quality assessment metric that measures the visual quality of an image. It evaluates the image quality by analyzing the texture and noise characteristics of the image.The lower the PIQE score, the better the image quality; UIQM usually combines the results of multiple dimensions such as brightness, contrast, and texture to give a composite score. The higher the UIQM score, the better the image quality.

Specifically, higher values of SSIM, PSNR, and UIQM indicate better image enhancement performance, while lower values of NIQE and PIQE reflect better image quality. Tables 1 and 2 compare the experimental results on Test1 and Test2 for eight selected models: WaterNet, UDCP, CLAHE, RGHS, HE, FUnIE-GAN, and the proposed model [23–28].

**Table 1:** Quantitative comparison of different methods on Test 1.

Model	SSIM	PSNR	NIQE	PIQE	UIQM
Input (Original)	N/A	N/A	4.12	41.23	2.37
WaterNet	0.77	22.02	3.51	15.46	2.59
UDCP	0.52	15.12	2.30	46.18	1.83
CLAHE	0.66	19.18	2.43	50.67	2.26
RGHS	0.70	23.14	2.31	47.12	2.08
HE	0.55	16.09	2.43	50.62	2.14
FUnIE-GAN	0.78	26.12	2.30	15.47	2.63
Ours	**0.81**	**26.89**	**2.27**	**15.23**	**3.12**

**Table 2:** Quantitative comparison of different methods on Test 2.

Model	SSIM	PSNR	NIQE	PIQE	UIQM
Input (Original)	N/A	N/A	3.93	39.73	2.88
WaterNet	0.78	22.21	2.49	16.10	2.62
UDCP	0.48	15.23	2.30	46.58	1.86
CLAHE	0.56	18.12	2.43	50.66	2.23
RGHS	0.72	23.16	2.31	45.13	2.12
HE	0.53	15.28	3.21	47.23	2.09
FUnIE-GAN	0.78	25.20	**2.36**	15.96	2.83
Ours	**0.79**	**26.23**	2.40	**15.62**	**3.11**

#### Qualitative evaluation results.

**Fig 6:**
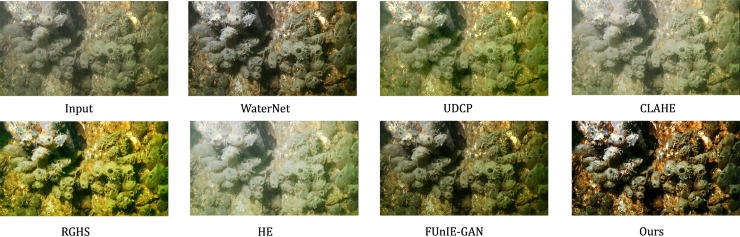
Image quality comparison of different models on the Test 1 test set.

**Fig 7:**
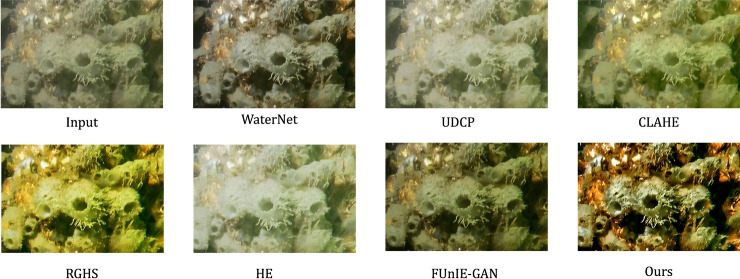
Comparison of image details of different models on the Test 1 test set.

Subjective analysis of the Test 1 sample images reveals that the original images exhibit degradation phenomena such as blurring and color distortion. After processing with the CLAHE and HE models, overexposure is commonly observed. Although the brightness of the images significantly increases, the overall structure is compromised, resulting in a loss of considerable details and a decline in image quality. The images enhanced by the RGHS and UDCP methods tend to have a yellow-green tint. Among these, the RGHS method performs better in terms of brightness enhancement, yielding visually superior results, while the images enhanced by UDCP show average brightness. The overall enhancement effects of the WaterNet and FUnIE-GAN models are better, with improved image clarity, but they remain relatively dark, and the brightness and contrast are still insufficient. In comparison with other methods, the technique proposed in this paper shows significant improvements in restoring the true colors of the image and substantially enhances the image contrast. This leads to a notable improvement in the visual quality of the processed images and a marked improvement in overall clarity, resulting in higher-quality images. The comparison of results and detailed images between the proposed method and other algorithms is shown in Figs 6 and 7.

**Fig 8:**
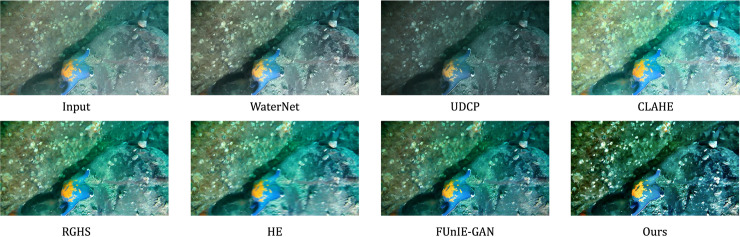
Enhanced images generated by different models on the Test 2 test set.

Subjective analysis of the Test 2 sample images indicates that the original images enhanced by WaterNet generally yield good results, but the details are relatively average. The images enhanced by UDCP are overall darker. The CLAHE method effectively improves the image clarity, achieving a relatively good enhancement, but the processed images show some color deviation compared to the original images. Both the RGHS and HE methods perform well, with RGHS being particularly strong in dehazing, while HE-enhanced images show average clarity, with some regions still appearing blurred. FUnIE-GAN excels in image enhancement, effectively improving clarity. However, images processed with this method suffer from lower overall brightness and insufficient contrast, which somewhat impacts the visual quality. In contrast, the method proposed in this paper not only effectively removes underwater haze but also provides superior overall clarity, better color balance, and the highest degree of color matching with the original images. Furthermore, this method outperforms other methods in terms of brightness and contrast and shows more refined detail handling. The enhanced images generated by each model on the Test 2 dataset are shown in Fig 8.

#### Ablation experiment.

**Fig 9:**
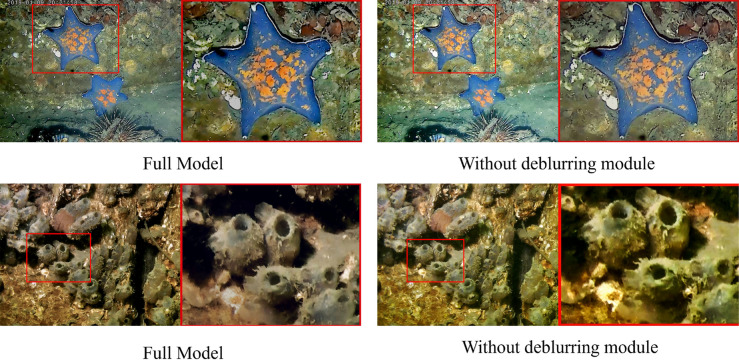
Qualitative ablation results of the proposed deblurring module.

In order to prove the effectiveness of the deblurring module, we set up two experimental groups to perform the task on real underwater images. Fig 9 illustrates the result for differernt models,where group A represents the model without deblurring module,and group B represents the independent function of defuzzification module is verified. We observed color enhancement inaccuracies in the images enhanced without deblurring Module,as the overall color of the image appeared yellowish and the edge details of the image were blurred. This verifies that deblurring model plays a crucial role in enhancing the overall color and texture details of the images.

It should be noted that the larger the PSNR value, the better the image quality; The larger the SSIM value, the better the image quality; UIQM is a no-reference underwater image quality assessment index based on the excitation of the human visual system, and the larger the value, the better the image quality. Table 3 shows the experimental results of the two groups on the dataset.

**Table 3:** Impact of deblurring modules on network performance.

Model	SSIM	PSNR	NIQE	PIQE	UIQM
A	0.76	26.12	2.34	27.43	2.91
B	0.81	26.89	2.27	15.23	3.12

## Conclusion

This paper adopts the diffusion model for unsupervised learning, combines the advantages of physical models and deep learning, and designs an underwater image enhancement algorithm that enhances the details of the image through multi-level design. The original image is processed in three steps. First, the image is decomposed using an encoder-decoder pipeline similar to the traditional U-Net structure, and then the initial enhanced image is extracted from the image through a color encoder network and adaptive color query is performed. Finally, the processed image is deblurred using the reverse denoising diffusion method to generate a clear underwater image. The results of the comparative experiment clearly show that compared with a variety of existing methods, the method proposed in this paper has significant advantages in eliminating the impact of water degradation on the image. The image enhanced by this method can more accurately reproduce the original features of the object, effectively improving the image quality and information restoration.
